# Nivolumab-Induced Neuromyopathy: A Case Report

**DOI:** 10.7759/cureus.69575

**Published:** 2024-09-17

**Authors:** Tal Sharon, Angela Rosenberg, Ekrem Yetiskul, Zaid Khamis, Suzanne El Sayegh

**Affiliations:** 1 Internal Medicine, Touro College of Osteopathic Medicine, New York, USA; 2 Internal Medicine, Staten Island University Hospital, Staten Island, USA; 3 Nephrology, Staten Island University Hospital, Staten Island, USA

**Keywords:** immune checkpoint inhibitor, neuromyopathy, nivolumab, oncology, squamous cell carcinoma of the head and neck

## Abstract

Nivolumab is an immune checkpoint inhibitor (ICI) that treats various malignancies. Although ICIs have proven efficacious, they can also have detrimental side effects. We present a case of nivolumab-induced quadriparesis mimicking Guillain-Barré syndrome in a patient with stage III squamous cell carcinoma (SCC) of the pharynx with a chronic tracheostomy, who presented after being found unconscious at home. He later developed acute kidney failure, requiring dialysis, and bilateral weakness of his upper and lower extremities. The patient was treated with corticosteroids and intravenous immunoglobulin (IVIG) with minimal improvement. Nivolumab-induced quadriparesis is very threatening and can be fatal if inappropriately managed. Therefore, we strongly advocate for a multidisciplinary team and early corticosteroid prescription to monitor patients on nivolumab therapy to prevent adverse clinical outcomes.

## Introduction

Nivolumab is a monoclonal antibody that binds to programmed cell death protein 1 (PD-1) on the surface of T-cells, blocking programmed cell death [1]. Programmed death-ligand 1 (PD-L1) is a receptor that can be expressed and upregulated by various cells, including tumor cells. Nivolumab works as an immune checkpoint inhibitor (ICI) to induce an anti-tumor immune response. It is approved for the treatment of numerous cancers, including squamous cell carcinoma (SCC) of the head and neck, non-small-cell lung cancer, melanoma, and Hodgkin lymphoma [1-3]. Notably, it has been shown to prolong survival in patients with head and neck cancer [2].

Despite the therapeutic efficacy of ICIs, their activating effects on the immune system generate the potential for unregulated immune responses. Consequently, ICIs may cause inflammatory toxicities in other tissues, leading to immune-related adverse events (irAEs). Neurological irAEs are infrequently discussed because they are rare, affecting <1% of patients [4,5], and more common in patients receiving anti-PD-1/PD-L1 inhibitors (6%) compared to those on anti-cytotoxic T-lymphocyte-associated protein-4 (CTLA-4) treatment (4%) [6]. In recent studies, the widely used immunotherapy has been associated with rapid-onset myopathies such as myasthenia gravis [6] and chronic inflammatory demyelinating polyradiculoneuropathy syndromes [7]. These have a median onset of four weeks from the commencement of therapy [4,5]. Meningitis, encephalitis, paraneoplastic syndromes, and peripheral neuropathies have also been seen, although rare as well [5].

We present a case of a 47-year-old male, previously functioning independently with a tracheostomy tube, on immunotherapy for stage III SCC of the pharynx, who rapidly developed quadriparesis and acute renal failure requiring dialysis, both adverse events to nivolumab.

## Case presentation

**Figure 1:**
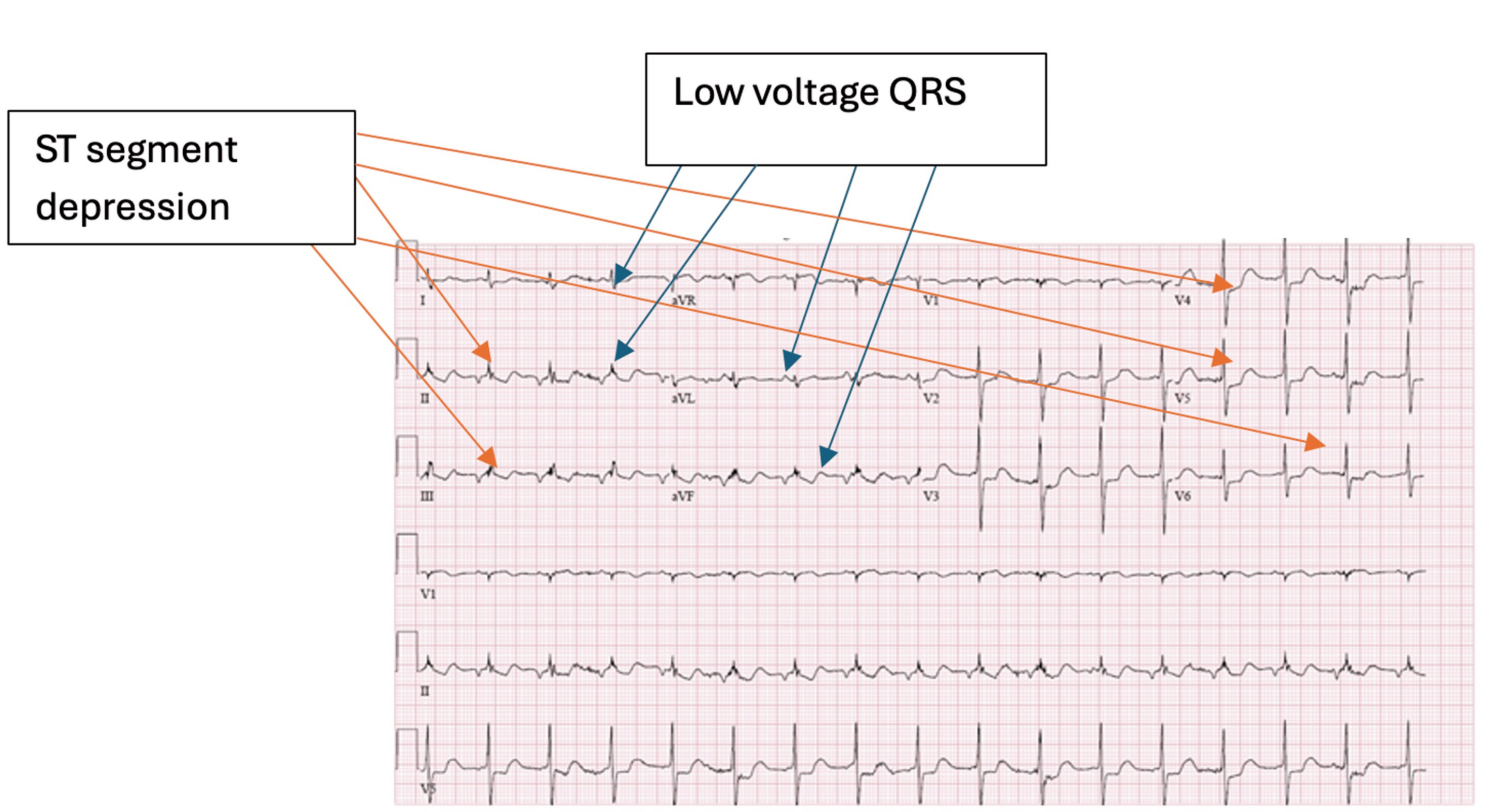
ECG demonstrating ST segment depression in inferolateral leads, low-voltage QRS complexes, and a short PR interval The ECG shows ST segment depression in the inferolateral leads (II, III, aVF, V4, V5, and V6). Additionally, the QRS complexes exhibit reduced amplitude across all leads, suggesting a low-voltage pattern. A short PR interval (100-120 msec) is also observed. ECG: electrocardiogram

A 47-year-old male with a history of stage III pharyngeal SCC, a chronic tracheostomy, and C2-C7 spinal fusion, was found unresponsive in his bathtub wearing four fentanyl patches. He last received cetuximab/nivolumabimmunotherapy one day prior. Emergency medical services noted that he was hypoxic, hypotensive, bradycardic, and exhibiting bradypnea with bloody emesis. Upon arrival at the emergency department, he presented with a fever of 102°F and a heart rate of 101 beats per minute. Laboratory evaluations (Table 1) were significant for a white blood cell count of 18.93 K/uL, creatine kinase of 7,612 U/L, creatinine of 2.4 mg/dL, troponin of 0.89 ng/mL, lactate of 15 mmol/L, and severe respiratory acidosis on venous blood gas. His electrocardiogram (ECG) showed a short PR interval and low-voltage QRS with ST depressions in the inferior leads (Figure 1). He was admitted to the intensive care unit (ICU) for septic shock due to pneumonia, rhabdomyolysis, non-ST-elevation myocardial infarction (NSTEMI), altered mental status, and possible drug overdose. While sedated, the patient had myoclonic jerking. An electroencephalogram (EEG) showed potential epileptiform activity in the left frontotemporal region. His myoclonic jerking was later attributed to toxic metabolic encephalopathy after routine EEG monitoring.

**Table 1:** Laboratory findings on admission WBC: white blood count, LDH: lactate dehydrogenase

Laboratory values	Reference ranges	Patient values
WBC	4.80-10.80 K/uL	18.93 K/uL
Platelets	130-400 K/uL	29 K/uL
Reticulocyte count	0.5%-1.5%	2.5%
Haptoglobin	34-200 mg/dL	135 mg/dL
Creatine kinase	0-225 U/L	7612 U/L
Creatinine	0.7-1.5 mg/dL	2.4 mg/dL
Troponin	≤0.01 ng/mL	0.89 ng/mL
Lactate	0.7-2 mmol/L	15 mmol/L
LDH	50-242 U/L	919 U/L

By day 3, the patient's rhabdomyolysis improved with IV fluids, although his creatinine levels increased, and he became oliguric. After an unsuccessful diuretics challenge, he required a Udall catheter and hemodialysis initiation. He developed severe thrombocytopenia, with platelets dropping to 29 K/uL. Potassium was monitored and kept within normal limits. Despite elevated lactate dehydrogenase (LDH) levels at 919 U/L, likely from rhabdomyolysis, tests for hemolysis, thrombotic thrombocytopenic purpura (TTP), and disseminated intravascular coagulation (DIC) were negative. By day 7, his myoclonus, transaminitis, and thrombocytopenia had resolved. He was moved to a step-down unit.

**Figure 2:**
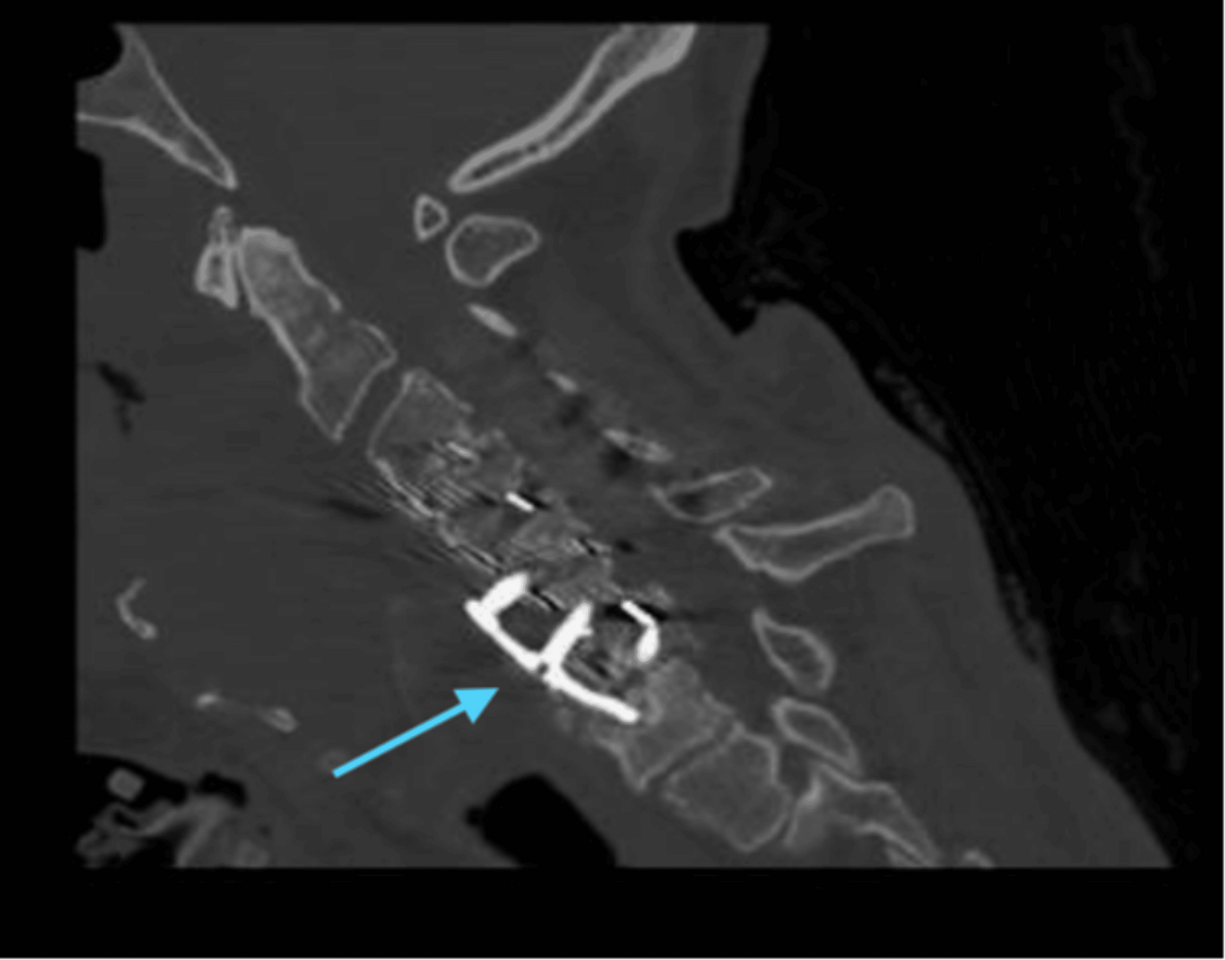
Coronal section obtained from CT of the patient's cervical spine demonstrates new compression fracture deformity of the C7 vertebral body (arrow) CT: computed tomography

On day 11, the patient experienced left-sided weakness after hemodialysis. No seizures were seen on video electroencephalogram (vEEG), no large vessel occlusion was seen on computed tomography angiogram (CTA) of the brain, and computed tomography perfusion studies were negative for ischemia. The stroke protocol workup was negative, but the patient remained weak. A course of methylprednisolone 60 mg every 12 hours was initiated to prevent further respiratory and renal decline and tapered down over the next week. The weakness progressed symmetrically, and on day 14, the patient could not move his arms or legs. A computed tomography (CT) of the cervical spine suggested a new compression fracture deformity of the C7 vertebral body (Figure 2), leading to severe stenosis with no recommended neurosurgery intervention. Six days after the onset of the hemiparesis, a lumbar puncture was performed to differentiate between a myelopathy and paraneoplastic syndrome.

After minimal improvements with corticosteroids, the patient began a five-day intravenous immunoglobulin (IVIG) course (0.4 gm/kg) for suspected Guillain-Barré syndrome, which marginally improved his neurological status. Despite regaining some finger and shoulder mobility, he remained paraplegic in the lower extremities and exhibited facial diplegia and a reduced gag reflex. Although ventilated and unable to speak, he was awake, alert, and maintained full sensory functions. Due to negligible recovery with corticosteroids or IVIG, he was administered a high-dose methylprednisolone (1 g daily for three days), followed by 60 mg of prednisone and a maintenance IVIG dose (0.5 g/kg every two weeks). The patient also experienced recurrent fevers with negative cultures.

All tested autoantibodies were negative, including gangliosides, angiotensin-converting enzyme, nuclear antigens, neutrophil cytoplasmic antigens, and anti-hu. Cerebrospinal fluid (CSF) analysis revealed elevated protein (261 mg/dL), albumin (269.9 mg/dL), and IgG (85.5 mg/dL) levels but normal glucose (60 mg/dL) and cell count (0/uL) (Table 2). He was diagnosed with ICI neuromyopathy, indicated by cytoalbuminologic dissociation.

**Table 2:** Cerebrospinal fluid studies CSF: cerebrospinal fluid

Laboratory values	Reference ranges	Patient values
IgG (CSF)	≤3.4 mg/dL	85.5 mg/dL
Albumin (CSF)	14-25 mg/dL	269.9 mg/dL
Glucose (CSF)	45-75 mg/dL	60 mg/dL
Protein (CSF)	15-45 mg/dL	261 mg/dL
Total nucleated cell count (CSF)	0-5 u/L	0 u/L

By day 31, the patient's creatinine stabilized at 0.5 mg/dL, eliminating the need for hemodialysis, although he continued experiencing unexplained fevers, peaking at 101.7°F. Despite receiving six IVIG doses by day 37, there was little motor strength improvement. On day 43, he developed a urinary tract infection caused by *Acinetobacter* and, despite antibiotic treatment, was transferred back to the ICU for increased vasopressor needs. He subsequently went into septic shock and was moved to his initial hospital for further rehabilitation.

## Discussion

The development of immune checkpoint inhibitors (ICIs) has been vital to cancer treatment. By inhibiting cytotoxic T-lymphocyte-associated protein-4 (CTLA-4) or programmed death protein-1 (PD-1), T-cells enhance the body's immune response to fighting cancer cells, protecting against autoimmunity, and simultaneously maintaining self-tolerance. The use of ICIs has become a standard of care in cancer therapy, marking a paradigm shift in treatment options for patients with advanced cancers [1-3].

Nivolumab, a monoclonal IgG4 antibody, is an ICI that binds to PD-1 to restore the balance of tumor-induced immunodeficiency. However, altering the immune system can create unregulated responses, leading to immune-related adverse events (irAEs). Often, these are manageable with corticosteroids. Rarely, they cause fatal events necessitating treatment discontinuation, threatening patient survival [4]. After a few rounds of immunotherapy, this patient presented with kidney injury requiring hemodialysis. Although he had normal-appearing kidneys on CT scan, his kidney function improved while on immunotherapy, and by the end of his hospital course, he no longer required hemodialysis. No kidney biopsy was obtained because it would not have changed management. The return of kidney function suggests a nivolumab-induced injury, although there is no direct evidence. Although most irAEs occur early during treatment [1], ICI-related neurotoxicity can occur within three months of initiation [5]. Nivolumab-induced irAEs affect all organ systems, causing colitis, pneumonitis, hypophysitis, and endocrine abnormalities [1-3]. Neurological irAEs are comparably infrequent manifestations and include Guillain-Barré syndrome, encephalitis, autoimmune neuropathy, and myasthenic syndrome [1]. Moreover, neurological irAEs are challenging to diagnose, often presenting with generalized symptoms such as fatigue and weakness, which could be natural effects of the cancer [7-9].

According to the American Society of Clinical Oncology, after stopping the ICI, corticosteroids are the mainstay of treatment for many irAEs, including those with neurological toxicity. Failure to improve on corticosteroids necessitates the initiation of plasmapheresis and IVIG [4,5]. In our case, administration of corticosteroids and IVIG both individually and concurrently resulted in minimal improvement. Negative autoantibody tests, coupled with the rapid onset of symptoms associated with nivolumab initiation and spontaneous resolution of kidney function with nivolumab elimination, indicated that our patient's toxicity was attributed to nivolumab therapy.

ICIs have expanded the landscape of treatment for advanced cancers. Although beneficial in diminishing inhibitory effects on T-cells and the immune system, the balance can become unregulated, fostering autoimmunity and toxicity [5]. In our case, the patient rapidly declined from the onset of his hospital admission, rendering him debilitated. Timely recognition of these fatal toxicities and early corticosteroid administration are imperative in preventing adverse outcomes. Monitoring kidney function (blood urea nitrogen (BUN)/creatinine (Cr)) could aid in the early identification of declining kidney function and prevent hemodialysis. While nerve conduction studies were not performed in this case, they could have been beneficial in identifying the specific nerves involved in the neuropathy, and therefore, we recommend it in this clinical context. A multidisciplinary team would also benefit patients on ICI therapy through monitoring for irAEs from initiation. Clinicians need to be aware of irAEs when prescribing immunotherapy, even those that are rare [4-6], as the potential for long-term morbidity and mortality is significant.

## Conclusions

Although ICIs have transformed the standard of care for various malignancies, they are a double-edged sword with many potentially devastating irAEs. In our case, the nivolumab-induced neuromyopathy and kidney dysfunction developed through an unknown mechanism and carried a poor prognosis. We strongly advise early corticosteroid administration to prevent potentially lethal irAEs. It would also be beneficial to monitor kidney function, using a multidisciplinary approach, to tackle any abnormalities. Appropriate recognition and timely management are vital in reducing morbidity and mortality associated with nivolumab reactions.
